# *GRIA3* Duplication at Xq25 in a Young Adult Male With Profound Intellectual Disability and Morbid Obesity: A Case Report

**DOI:** 10.1155/crig/7670059

**Published:** 2026-09-22

**Authors:** Shuichi Yatsuga

**Affiliations:** ^1^ Department of Pediatrics, Fukuoka University, Fukuoka, Japan, fukuoka-u.ac.jp; ^2^ Department of Medical Genetics, Hakodate Goryoukaku Hospital, Hakodate, Hokkaido, Japan

**Keywords:** autism spectrum disorder, case report, chromosomal microarray analysis, *GRIA3*, intellectual disability, obesity, valproate, Xq25 duplication

## Abstract

We report a 22‐year‐old Japanese male with profound intellectual disability (IQ 16), autism spectrum disorder (ASD), and drug‐controlled epilepsy referred for obesity management (height 182.7 cm, weight 139.7 kg, and BMI 41.9 kg/m^2^). Clinical features included brachycephaly, deep‐set eyes, prominent supraorbital ridges, bitemporal narrowing, broad nasal root, hypotonia, hyporeflexia, and sleep–wake cycle disorder. Chromosomal microarray analysis (CMA) performed by SRL Inc. (Tokyo, Japan) using an Affymetrix‐based platform (specific array model not disclosed by the laboratory) identified a duplication at Xq25 (minimum interval: chrX:121,515,588–122,689,238; maximum interval: chrX:121,468,613–122,733,639; GRCh37/hg19; arr[GRCh37] Xq25 (121,515,588_122,689,238) × 2) encompassing the entire *GRIA3* gene, classified as a variant of uncertain significance (VUS). The duplicated interval did not encompass *STAG2*. Parental genetic testing was declined and exome or genome sequencing was not performed; an alternative molecular diagnosis cannot be excluded. Comorbid findings included Grade 2 hypertension (161/99 mmHg), hemoglobin elevation (17.2 g/dL) possibly consistent with sleep‐disordered breathing (not formally evaluated), hepatic steatosis on ultrasonography, splenomegaly, and hyperuricemia. Progressive weight gain had begun at approximately 9 years of age, preceding antipsychotic and antiepileptic polypharmacy by approximately 8 years. Dietary and physical activity guidance without pharmacotherapy resulted in an approximately 4‐kg weight reduction over 4 months (BMI 40.6 kg/m^2^). This case describes a range of metabolic findings in a patient carrying a GRIA3 duplication.

## 1. Introduction

*GRIA3*, located at Xq25, encodes glutamate receptor ionotropic AMPA subunit 3 (GluA3), a component of AMPA‐type glutamate receptors (AMPARs) that mediate excitatory synaptic transmission. *GRIA3* is X‐linked, and pathogenic variants have been reported predominantly in males [1]. Both gain‐of‐function and loss‐of‐function *GRIA3* variants have been described; loss‐of‐function variants tend to be associated with older seizure onset, hypotonia, and sleep disturbances, while gain‐of‐function variants cause earlier and more severe epileptic phenotypes [1]. Characteristic features include brachycephaly, prominent supraorbital ridges, deep‐set eyes, and bitemporal narrowing [2, 3]. Other reported features include intellectual disability, epilepsy, ASD, hyporeflexia, sleep–wake cycle disorder, and self‐injurious behavior [2–5].

Duplications at Xq25 involving *GRIA3* have been described in males with syndromic intellectual disability [3, 6, 7]. In some cases, the duplicated interval also encompassed the adjacent *STAG2* gene [3]; in others, partial intragenic duplications of *GRIA3* were associated with aberrant transcripts leading to reduced AMPA receptor expression [6, 7]. Here, we describe a patient with a duplication at Xq25 encompassing the entire *GRIA3* gene, whose predominant clinical concern at the time of referral was morbid obesity.

## 2. Case Presentation

### 2.1. Clinical History and Presentation

The patient is a 22‐year‐old Japanese male (born October 2003) referred to the clinical genetics outpatient service for management of severe obesity. He was the second of dichorionic–diamniotic twins, born at 37 weeks of gestation by cesarean section (birth weight 2410 g, body length 45.5 cm, and head circumference 31.5 cm). His co‐twin brother has a normal phenotype (height 179 cm and weight approximately 70 kg), as do both parents and two younger brothers. Language development was delayed beyond 2 years of age. At 2 years and 9 months, he was diagnosed with ASD. Self‐injurious behavior (head‐banging) was first noted at 2 years of age and was ongoing at the time of referral; aripiprazole was subsequently initiated for behavioral management including self‐injury. He attended special education and graduated high school. Intellectual testing at age 17 using the Tanaka‐Binet Intelligence Scale (a standardized test validated for the Japanese population) yielded an IQ of 16, consistent with profound intellectual disability. He currently attends a supported employment B‐type program. Epilepsy was first diagnosed at age 18 following an episode of loss of consciousness; seizures have been controlled since approximately age 19. Progressive weight gain began at approximately 9 years of age. Aripiprazole was first initiated at approximately 17 years of age, and sodium valproate and lamotrigine at approximately 18 years of age (following epilepsy diagnosis); weight gain therefore preceded antipsychotic and antiepileptic polypharmacy by approximately 8 years. Sleep–wake cycle disturbance, characterized by nocturnal awakening and difficulty maintaining sleep, was reported by family members; ramelteon had been prescribed for this at the referring clinic. Current medications include aripiprazole 24 mg/day, sodium valproate 200 mg/day, lamotrigine 300 mg/day, ramelteon 8 mg nightly, and diazepam 5 mg as needed on weekends.

### 2.2. Physical Examination

At first visit, height was 182.7 cm, weight was 139.7 kg (BMI 41.9 kg/m^2^), body fat percentage was 40.9%, and muscle mass was 78.4 kg. Blood pressure was 161/99 mmHg (Grade 2 hypertension; not previously evaluated). Facial features included brachycephaly, prominent supraorbital ridges, deep‐set eyes, bitemporal narrowing, and broad nasal root, consistent with features reported in previously described males with Xq25 duplications [3]. Abdominal striae and acanthosis nigricans of the neck were present. Snoring was reported by family members, and lower‐limb cyanosis was observed in the sitting position. Hypotonia and hyporeflexia were present on neurological examination.

### 2.3. Laboratory and Imaging Findings

Clinical and laboratory findings are summarized in Table 1. Laboratory findings included hemoglobin elevation (17.2 g/dL) and hematocrit elevation (53.1%), which may be consistent with secondary erythrocytosis; sleep‐disordered breathing was clinically suspected based on snoring and sitting‐position cyanosis, but was not formally evaluated, and other causes of erythrocytosis were not excluded. Additional abnormalities included elevated liver enzymes (ALT 89 U/L, γ‐GTP 99 U/L), hyperuricemia (uric acid 8.3 mg/dL), mildly elevated CRP (0.45 mg/dL), low serum IGF‐1 (somatomedin C 124 ng/mL), and mild hypophosphatemia (inorganic phosphorus 1.7 mg/dL). Fasting glucose was 120 mg/dL and HbA1c 5.7%, indicating impaired fasting glucose. Thyroid function, serum ACTH, and cortisol were within normal limits. Abdominal ultrasonography demonstrated hepatic steatosis (increased echogenicity with hepato‐renal contrast enhancement and posterior acoustic attenuation) and mild splenomegaly (long axis 143 mm). Formal assessment of hepatic fibrosis was not performed. Formal polysomnography and brain MRI were not performed due to behavioral constraints.

**TABLE 1:** Summary of clinical and laboratory findings.

Parameter	Finding
Age/sex	22 years/male
Height/weight/BMI	182.7 cm/139.7 kg/41.9 kg/m^2^
Weight at 4 months	135.4 kg (BMI 40.6 kg/m^2^)
Blood pressure	161/99 mmHg (Grade 2 hypertension; first documented)
Diagnoses	Profound intellectual disability (IQ 16), ASD, drug‐controlled epilepsy, morbid obesity
CMA (Affymetrix‐based; SRL Inc., Tokyo)	arr[GRCh37] Xq25 (121,515,588_122,689,238) × 2; entire *GRIA3* encompassed; *STAG2* NOT included (∼360 kb distal); orientation unknown; VUS; parental testing declined
Facial features	Brachycephaly, prominent supraorbital ridges, deep‐set eyes, bitemporal narrowing, broad nasal root
Neurological findings	Hypotonia, hyporeflexia, sleep‐wake cycle disorder, self‐injurious behavior (head‐banging since age 2)
Hemoglobin/hematocrit	17.2 H g/dL/53.1 H % (possible erythrocytosis; etiology not confirmed)
ALT/γ‐GTP	89 H/99 H (U/L)
Uric acid	8.3 H mg/dL
Fasting glucose/HbA1c	120 H mg/dL/5.7% (impaired fasting glucose)
IGF‐1 (somatomedin C)	124 L ng/mL
Inorganic phosphorus	1.7 L mg/dL
Cortisol/ACTH/thyroid	Within normal limits
Abdominal US	Hepatic steatosis (+); splenomegaly (143 mm); no cirrhotic changes on imaging
Sleep study/brain MRI	Not performed (behavioral constraints)
Additional genetic testing	Exome/genome sequencing not performed
Medications	Valproate 200 mg, lamotrigine 300 mg, aripiprazole 24 mg, ramelteon 8 mg, diazepam 5 mg PRN
Weight gain onset	∼Age 9 (∼2012); polypharmacy initiated ∼age 17–18 (2021)
Intervention	Dietary guidance 1800 kcal/day + physical activity; GLP‐1 agonist not initiated

### 2.4. Genetic Findings

CMA was performed by SRL Inc. (Tokyo, Japan) using an Affymetrix‐based platform (specific array model not disclosed by the laboratory). A duplication at Xq25 was identified: minimum interval chrX:121,515,588–122,689,238; maximum interval chrX:121,468,613–122,733,639 (GRCh37/hg19). ISCN 2020 nomenclature: arr[GRCh37] Xq25 (121,515,588_122,689,238) × 2 (minimum interval). The duplicated segment spans approximately 1.17–1.27 Mb and encompasses the entire *GRIA3* gene (chrX:122,318,131–122,624,766; approximately 307 kb; plus strand). The duplicated interval also includes approximately 800 kb of sequence upstream and 109 kb downstream of *GRIA3*. Within the duplicated interval, *GRIA3* is the only annotated protein‐coding gene with an established disease association; the upstream flanking sequence is a gene‐poor region containing no annotated protein‐coding genes with a known Mendelian disease association. Three genes previously implicated in Xq25 duplication syndrome lie outside the duplicated interval: *THOC2* (chrX:122,734,412–122,866,906), which begins approximately 0.8 kb distal to the maximum interval boundary; *XIAP*; and *STAG2*. The duplicated region ends approximately 360 kb proximal to *STAG2* (chrX:123,094,062–123,556,514), which lies outside the duplicated interval. The variant is classified as VUS because (a) parental genetic testing was declined (inheritance pattern unknown); (b) no functional study was performed; and (c) no structurally identical variant (a whole‐gene *GRIA3* duplication without additional co‐duplicated genes and with confirmed pathogenicity) has been reported in the literature with formal segregation data. A Y‐chromosome variant was classified as benign. CMA has historically been recommended as a first‐tier diagnostic test for unexplained intellectual disability and ASD [8]; current guidelines now recommend exome or genome sequencing as a first‐ or second‐tier test in this clinical setting [9]. Exome or genome sequencing was not performed, and an alternative molecular diagnosis contributing to the patient’s neurodevelopmental phenotype cannot be excluded.

The duplication encompasses the entire *GRIA3* gene. This is structurally distinct from the partial intragenic *GRIA3* duplications reported by Chiyonobu et al. [6] and Bonnet et al. [7]. The orientation of the duplicated segment (tandem, inverted, or other) is unknown, as CMA does not resolve structural configuration; FISH or long‐read sequencing was not performed. Parental genetic testing (FISH/MLPA) was declined by the family, and twin zygosity was not determined. The patient’s phenotype—including profound intellectual disability, ASD, epilepsy, characteristic facial features, hypotonia, hyporeflexia, sleep–wake cycle disorder, and self‐injurious behavior—is consistent with features described in males with Xq25 duplications involving *GRIA3* [2–5], though a causal relationship between the VUS and the phenotype cannot be formally established.

### 2.5. Management and Outcome

Pharmacological intervention with a GLP‐1 receptor agonist was discussed with the family but was not initiated. Dietary intervention targeting 1800 kcal/day was provided by a registered dietitian, with guidance sessions for the patient and family. Physical activity guidance encouraged family‐supported walking. Over 4 months, weight decreased from 139.7 kg to approximately 135.4 kg (BMI 40.6 kg/m^2^), a reduction of approximately 4 kg.

## 3. Discussion

The patient described here carries a duplication at Xq25 encompassing the entire *GRIA3* gene. His neurodevelopmental features—profound intellectual disability, ASD, drug‐controlled epilepsy, brachycephaly, prominent supraorbital ridges, deep‐set eyes, bitemporal narrowing, broad nasal root, hypotonia, hyporeflexia, sleep–wake cycle disorder, and self‐injurious behavior—are features that have been described in males with Xq25 duplications involving *GRIA3* [2–5]. The relationship between the identified VUS and this phenotype cannot, however, be established with certainty on the basis of CMA alone, given the absence of parental testing, functional data, and exome or genome sequencing.

This is structurally different from the partial intragenic *GRIA3* duplications described by Chiyonobu et al. [6] and Bonnet et al. [7], in which aberrant transcripts with premature termination codons were directly demonstrated by expression analysis. In the present case, the orientation of the duplicated segment is unknown, and the functional consequences—which might include increased gene dosage, position effects, altered regulatory interactions, or no functional change—cannot be determined from CMA alone. The most structurally comparable published cases remain those of Philippe et al. [3], in which Xq25 duplications encompassing both *GRIA3* and *STAG2* were associated with neurodevelopmental phenotypes; the present case differs in that *STAG2* is not included in the duplicated interval.

Morbid obesity (BMI 41.9 kg/m^2^) was the primary reason for referral. Progressive weight gain began at approximately 9 years of age, approximately 8 years before the initiation of antipsychotic (aripiprazole, age 17) and antiepileptic (valproate, lamotrigine, age 18) polypharmacy. ASD‐related behavioral hyperphagia and limited physical activity from early childhood are therefore likely primary contributors to the onset of obesity, while subsequent polypharmacy—valproate and aripiprazole are both associated with weight gain [10, 11]—may have contributed to further weight accumulation over time. A direct genotype–obesity relationship cannot be established.

The metabolic comorbidities identified—impaired fasting glucose, hepatic steatosis, hyperuricemia, and hypertension (161/99 mmHg, first documented at this visit)—are consistent with obesity‐associated findings. Hemoglobin elevation (17.2 g/dL, hematocrit 53.1%) may be consistent with secondary erythrocytosis from sleep‐disordered breathing, which was clinically suspected based on snoring and sitting‐position cyanosis; however, formal polysomnography was not performed and other causes were not excluded.

Mild splenomegaly (143 mm) was noted. Hepatic steatosis was present on ultrasonography, but cirrhotic changes were not observed; formal fibrosis assessment was not performed. The cause of splenomegaly was not determined. Mild hypophosphatemia may reflect valproate‐associated proximal tubular dysfunction, vitamin D deficiency in the context of obesity, or nutritional factors related to food selectivity; further evaluation was not performed within the observation period. Serum IGF‐1 was low, a finding consistent with obesity‐related suppression of the GH‐IGF axis.

Dietary and physical activity guidance without pharmacotherapy resulted in an approximately 4‐kg weight reduction over 4 months, suggesting that structured lifestyle intervention may produce modest weight reduction in patients with profound intellectual disability and ASD when supported by a multidisciplinary team and family involvement. The longer‐term sustainability of this change was not assessed.

## Funding

No external funding was received.

## Ethics Statement

This case report was conducted in accordance with the Declaration of Helsinki. Written informed consent for publication was obtained from the patient’s legal guardian (mother) on 19 May 2026 (Wiley Patient Consent Form).

## Conflicts of Interest

The author declares no conflicts of interest.

## Supporting Information

Additional supporting information can be found online in the Supporting Information section.

## Supporting information


**Supporting Information** A completed CARE checklist is available as Supporting Information.

## Data Availability

The data that support the findings of this study are available from the corresponding author upon reasonable request.
