# Supplementary material for: GRIA3 Duplication at Xq25 in a Young Adult Male With Profound Intellectual Disability and Morbid Obesity: A Case Report
Source: Case Rep Genet. 2026 Sep 22;2026:7670059. doi: 10.1155/crig/7670059 (PMC13595809; doi:10.1155/crig/7670059)
Supplement: Supplementary file 1 [file CRIG-2026-7670059-s001.docx]

**CARE Checklist — Revised Version**

*Manuscript No. 7670059 | Case Reports in Genetics*

*Title: GRIA3 duplication at Xq25 in a young adult male with profound intellectual disability and morbid obesity: a case report*

Author: Shuichi Yatsuga | Revised: August 28, 2026 (2nd revision)

| **Item** | **Sub-item** | **Checklist Item Description** | **Present? / Location in Manuscript** |
| --- | --- | --- | --- |
| **1. Title** | | | |
| 1 | — | The diagnosis or intervention of primary focus followed by the words "case report" | **Yes** Title page: '…morbid obesity: a case report' |
| **2. Key Words** | | | |
| 2 | — | 2 to 5 key words that identify diagnoses or interventions in this case report, including "case report" | **Yes** Title page: 8 keywords: GRIA3, Xq25 duplication, intellectual disability, autism spectrum disorder, obesity, valproate, chromosomal microarray analysis, **case report** |
| **3. Abstract** | | | |
| 3 | 3a | Introduction — What is unique about this case? | **Yes** Abstract: GRIA3 whole-gene duplication (unknown orientation) with morbid obesity; STAG2 not encompassed |
| 3 | 3b | Patient information — Key demographics and presenting concerns | **Yes** Abstract: 22-year-old male; profound ID; ASD; BMI 41.9 kg/m² |
| 3 | 3c | Clinical findings — Main symptoms and important clinical findings | **Yes** Abstract: dysmorphic features, hypertension, hemoglobin elevation (etiology not confirmed), hepatic steatosis, splenomegaly, hyperuricemia |
| 3 | 3d | Diagnoses — Assessment and diagnostic results | **Yes** Abstract: CMA (Affymetrix-based; SRL Inc.) — ISCN arr[GRCh37] Xq25(121,515,588_122,689,238)×2; entire GRIA3 encompassed; orientation unknown; VUS; exome/genome sequencing not performed |
| 3 | 3e | Interventions — Primary interventions | **Yes** Abstract: dietary guidance (1,800 kcal/day) + physical activity; GLP-1 receptor agonist discussed with family but not initiated |
| 3 | 3f | Outcomes — Including adverse events | **Yes** Abstract: ~4 kg weight reduction over 4 months; no adverse events from intervention |
| 3 | 3g | Conclusion — Main take-away lesson | **Yes** Abstract: range of metabolic findings described; lifestyle intervention feasible |
| 3 | 3h | Informed consent — Was consent obtained? | **Yes** Declarations: Wiley Patient Consent Form signed by mother (legal guardian), 19 May 2026 |
| **4. Introduction** | | | |
| 4 | — | Briefly summarize why this case is unique with medical literature references | **Yes** Section 1: GRIA3-related disorder overview; whole-gene duplication without STAG2; morbid obesity not previously described in this context; refs [1-7] |
| **5. Patient Information** | | | |
| 5 | 5a | De-identified patient demographic information | **Yes** Section 2.1: 22-year-old Japanese male; twin (zygosity undetermined); supported employment B-type program; identity protected |
| 5 | 5b | Main concerns and symptoms | **Yes** Section 2.1: morbid obesity (BMI 41.9 kg/m²) as primary referral reason; neurodevelopmental features |
| 5 | 5c | Medical, family, and psychosocial history including genetic information | **Yes** Section 2.1: ASD (2y9mo), epilepsy (18y), self-injurious behavior (head-banging from age 2), sleep-wake cycle disorder (nocturnal awakening, ramelteon prescribed); weight gain from age 9, preceding polypharmacy by ~8 years; family history negative for NDD; co-twin unaffected; genetic: GRIA3 Xq25 duplication (VUS); exome/genome not done |
| 5 | 5d | Relevant past interventions and their outcomes | **Yes** Section 2.1: aripiprazole (age 17), valproate + lamotrigine (age 18), ramelteon; no prior obesity pharmacotherapy |
| **6. Clinical Findings** | | | |
| 6 | — | Relevant physical examination and significant clinical findings | **Yes** Section 2.2: height/weight/BMI; BP 161/99 mmHg (first documented); facial dysmorphism; acanthosis nigricans; abdominal striae; hypotonia; hyporeflexia; lower-limb cyanosis (sitting position) |
| **7. Timeline** | | | |
| 7 | — | Historical and current information organized as a timeline | **Yes** Embedded in Section 2.1. Key events: birth 2003 → ASD 2y9mo → self-injury onset 2y → weight gain onset ~9y → aripiprazole ~17y → epilepsy/valproate ~18y → seizure control ~19y → first visit Feb 2026 → CMA result Feb 2026 → abdominal US Apr 2026 → 4-month follow-up Jun 2026 |
| **8. Diagnostic Assessment** | | | |
| 8 | 8a | Diagnostic methods | **Yes** Section 2.3–2.4: CMA (Affymetrix-based; SRL Inc.; ISCN provided; gene content of interval stated); blood tests; abdominal US; body composition (InBody); BP |
| 8 | 8b | Diagnostic challenges | **Yes** Section 2.3–2.4: polysomnography and brain MRI not performed (behavioral constraints); parental testing declined; twin zygosity undetermined; EEG/seizure data unavailable (managed at referring institution); exome/genome sequencing not performed |
| 8 | 8c | Diagnostic reasoning including differential diagnosis | **Yes** Section 2.4 & 3: VUS classification rationale explicitly stated (no parental data, no functional study, no structurally identical variant with confirmed pathogenicity); secondary causes of obesity excluded (Cushing, hypothyroidism); hemoglobin elevation — sleep-disordered breathing suspected but unconfirmed; alternative molecular diagnoses not excluded; GRIA3 only disease-associated gene in interval, THOC2/XIAP/STAG2 excluded |
| 8 | 8d | Prognostic characteristics | **N/A** Not applicable |
| **9. Therapeutic Intervention** | | | |
| 9 | 9a | Types of intervention | **Yes** Section 2.5: dietary intervention (1,800 kcal/day) + physical activity guidance; GLP-1 receptor agonist (discussed with family, not initiated) |
| 9 | 9b | Administration of interventions | **Yes** Section 2.5: dietitian-led sessions for patient and family; target 1,800 kcal/day; family-supported walking; 4-month period |
| 9 | 9c | Changes in interventions | **Yes** Section 2.5: no changes during observation period |
| **10. Follow-up and Outcomes** | | | |
| 10 | 10a | Clinician-assessed outcomes | **Yes** Section 2.5: weight 139.7 kg → 135.4 kg over 4 months (BMI 41.9 → 40.6 kg/m²) |
| 10 | 10b | Patient-reported outcomes | **N/A** Not formally assessable (profound ID; IQ 16; severe communication impairment) |
| 10 | 10c | Adverse and unanticipated events | **Yes** Section 2.5: no adverse events from dietary or activity intervention |
| **11. Discussion** | | | |
| 11 | 11a | Strengths and limitations | **Yes** Section 3: VUS (no parental testing, no functional study, no exome/genome); orientation of duplication unknown; polysomnography/MRI not performed; seizure/EEG data unavailable; twin zygosity unknown; FIB-4 not used (not validated in age 22) |
| 11 | 11b | Discussion of relevant medical literature | **Yes** Section 3: Rinaldi 2024, Trivisano 2020, Philippe 2013, Chiyonobu 2007, Bonnet 2009; Verrotti 2011, Dayabandara 2017 for polypharmacy-related weight gain |
| 11 | 11c | Rationale for conclusions | **Yes** Section 3: whole-gene GRIA3 duplication (not STAG2); VUS limitations explicitly acknowledged; weight gain preceding polypharmacy stated; obesity causality not attributed to GRIA3 |
| 11 | 11d | Primary take-away lesson | **Yes** Section 3 final paragraph: structured lifestyle intervention without pharmacotherapy may produce modest weight reduction in patients with profound ID and ASD when supported by a multidisciplinary team; long-term sustainability of this outcome was not assessed. |
| **12. Patient Perspective** | | | |
| 12 | — | Patient's perspective on treatment whenever possible | **N/A** Not obtainable. Patient has profound ID (IQ 16) and severe communication impairment. Family (mother, legal guardian) provided information throughout. |
| **13. Informed Consent** | | | |
| 13 | — | Did the patient give informed consent? | **Yes** Declarations: Wiley Patient Consent Form signed by mother (legal guardian, Yuko Akimoto) on 19 May 2026. Original retained by author. |

*Revision note (2nd revision): Updated in response to Reviewer 1 minor comments — gene content of the duplicated interval is now stated in Section 2.4 (GRIA3 is the only protein-coding gene in the interval with an established disease association; THOC2, XIAP and STAG2 lie outside it), and the CMA first-tier statement has been reworded as a historical recommendation with a current guideline reference added [11].*
